# Early life adversity shapes neural and behavioural responses to oxytocin during altruistic decision-making

**DOI:** 10.1038/s41398-026-04302-0

**Published:** 2026-07-27

**Authors:** Nina Marsh, Vanessa Jeske, Mari Babasiz, Angela Herscheid, Ann-Kathrin Kreuder, Rüdiger Stirnberg, Tony Stoecker, Abigail A. Marsh, Johannes Schultz, René Hurlemann

**Affiliations:** 1 Department of Psychiatry & Psychotherapy, School of Medicine & Health Sciences, Carl von Ossietzky University of Oldenburg, Oldenburg, Germany; 2 Department of Orthopedics and Trauma Surgery, University of Bonn, Bonn, Germany; 3 Department of Psychiatry and Psychotherapy, Correctional Facility Plötzensee, Berlin, Germany; 4 Division of Medical Psychology, Department of Psychiatry and Psychotherapy, University Hospital Bonn, Bonn, Germany; 5 Department of MR Physics, German Center for Neurodegenerative Diseases, Bonn, Germany; 6 Department of Physics and Astronomy, University of Bonn, Bonn, Germany; 7 Department of Psychology, Georgetown University, 3700 O St NW, Washington, DC USA; 8 Interdisciplinary Program in Neuroscience, Georgetown University, 3700 O St NW, Washington, DC USA; 9 Center for Economics and Neuroscience, University of Bonn, Bonn, Germany; 10 Institute of Experimental Epileptology and Cognition Research, Medical Faculty, University of Bonn, Bonn, Germany; 11 Research Center Neurosensory Science, University of Oldenburg, Oldenburg, Germany

**Keywords:** Neuroscience, Human behaviour

## Abstract

What makes some people willing to help strangers at a personal cost, while others are more hesitant or selective? Early life adversity - such as neglect or abuse - may shape how we respond to strangers in need later in life, yet the underlying neural mechanisms remain unclear. In this study we tested whether the neuropeptide oxytocin, a key modulator for a rich repertoire of social behaviours, influences altruistic decisions in adults with different childhood experiences. Using functional magnetic resonance imaging (fMRI) and a validated altruistic donation task, the study showed that oxytocin increased donations among individuals with greater childhood adversity, while reducing them in those with lower adversity. These behavioural effects were mirrored by changes in functional connectivity between the medial prefrontal cortex and middle cingulate cortex - core circuits involved in social evaluation and empathy. Our findings suggest that early life experiences sensitize neural pathways for social processing, and that oxytocin dynamically modulates these circuits to influence altruistic behaviour in adulthood.

## Introduction

Why do some individuals engage in acts of cooperation, generosity, and helping, even at personal cost, while others are more hesitant or selective? Understanding the neurobiological and experiential factors that underlie this variation is especially important in light of evidence that early adversity—such as childhood maltreatment—can lead to long-term reductions in prosociality [1]. This raises a crucial question: Can a social neuromodulator like oxytocin (OT) temporarily restore altruistic tendencies in those affected? Childhood adversity (CA), including physical and emotional abuse, has been linked to enduring changes in social cognition and behaviour. CA is associated with heightened sensitivity to social threat and ambiguity [2, 3], increased antisocial behaviour [4], and difficulties in forming close interpersonal bonds [5], suggesting that it may play a critical role in individual differences in altruism. Although much research has focused on its effects on internalizing and externalizing problems, less attention has been paid to the implications of CA on prosocial outcomes such as altruistic donations [1]. Yet, given that more than half of adults worldwide report at least one adverse childhood experience [6], understanding how CA shapes prosocial behaviour is of both theoretical and practical importance.

Identifying the neurobiological pathways that could account for or temporarily induce a shift toward increased prosocial responding following CA is critical for mitigating its long-term effects on both individuals and communities. One promising candidate is OT, a hypothalamic neuropeptide that modulates a broad repertoire of social behaviours - including trust, empathy, interpersonal behaviour, and altruism - through both central and peripheral pathways [7–9] in a person- and context-dependent manner [10, 11]. The peptide influences sensitivity to social cues [12, 13] and tends to promote social approach behaviours [9, 14]. This neuromodulatory profile of OT is further supported by neuroimaging studies showing that OT consistently targets reward- [15, 16] and fear-related brain circuits [17, 18]. Importantly, recent meta-analytic findings indicate that individuals exposed to early adversity show reduced baseline OT levels across development [19] and decreased OT receptor expression in regions such as the cingulate cortex [20]. Such alterations may reduce social approach motivation and impair prosocial behaviour, but how such reduced baseline OT levels implicate (pro)social behaviour remains elusive. One possibility is that OT exerts the strongest effects in those with lower baseline altruism - consistent with theories positing an inverted U-shaped dose–response function [21, 22], in which both hypo- and hyper-activation of the OT system may fail to produce prosocial outcomes. In this context, individual experiences of CA may critically shape how OT influences altruistic behaviour, potentially by altering baseline social dispositions or sensitivity to social cues.

This study aimed to determine whether OT can partially restore diminished prosocial behaviour in adults with higher levels of CA. First, given the link between CA and reduced altruism, we hypothesized that participants with higher CA exposure would show lower altruistic behaviour in a validated donation task compared to participants with less CA exposure. Second, based on the potential of OT to enhance prosocial behaviour in vulnerable populations, we predicted that OT would have different effects on altruistic behaviour depending on participants’ CA exposure. In this context, the neural mechanisms underlying potential OT effects during altruistic donations might engage regions implicated in empathy and prosocial responses to distress and pain [20, 23], so OT may modulate prosocial decision-making through effects on this broader neural network. We tested these hypotheses using high-resolution functional magnetic resonance imaging (7-Tesla fMRI) in a randomized, placebo-controlled, double-blind, between-group study involving 54 healthy male participants. To our knowledge, this is the first study to map the neural effects of intranasal OT during an altruistic donation task in the context of CA, providing novel insights into the neurobiological plasticity of prosociality.

## Materials and methods

### Experimental design

We performed a randomized, double-blind, placebo-controlled, between-group design study. A total of 61 subjects were randomly assigned to either the intranasal administration OT (24 IU; six puffs per nostril each with 2 IU; Novartis) or PLC (24 IU; six puffs per nostril each with 2 IU containing the identical ingredients except the peptide). Seven participants were excluded from data analysis due to technical issues (n = 1), anomalies in the MRI scan (n = 2), or non-compliance with the task instruction, as indicated by donation responses to more than five neutral items (n = 4). Thus, we performed the final fMRI data analysis with a total of 54 subjects: 27 in the PLC group and 27 in the OT group. An a priori power analysis was conducted for the overarching project using G*Power 3 [24], based on effect sizes obtained in a prior OT dose-response study [22] (dz = 0.56; converted to *d* = 0.78 for a between-subject design). This analysis indicated that at least 48 participants would be required to detect an OT effect (α = 0.05, power = 0.75). The MRI task took place 40–45 min after the administration of the nasal spray. This timing was chosen in line with the expected peak central effects of intranasal OT, as suggested by prior pharmacokinetic and neuroimaging studies [22, 25], indicating maximal central effects approximately 30–50 min after administration. Accordingly, task performance in this study was scheduled within this timeframe to capture peak behavioural and neural effects of OT. Given inconsistent evidence in the literature regarding a change in endogenous OT levels in individuals with a history of childhood maltreatment [26, 27], endogenous OT is not a reliable biomarker according to current knowledge. For this reason, we did not measure endogenous OT levels.

### Participants

A total of 61 healthy males (mean age 25.20 ± 4.12 years) with no current or past physical or psychiatric illness participated in this study after giving written informed consent. Subjects were free of current and past physical or psychiatric illness, as assessed by medical history and the Mini-International Neuropsychiatric Interview (MINI) [28]. The Childhood Trauma Questionnaire (CTQ) [29], which is one of the most widely applied instruments to assess CA, was used to explore the association between CA and altruistic behaviour in this study. To control for possible pre-treatment effects, we assessed anxiety traits with the State-Trait Anxiety Inventory [30], depressive symptoms with the Beck Depression Inventory [31], and autistic-like traits with the Autism-Spectrum-Quotient [32]. Furthermore, we assessed cooperative and altruistic attitudes based on the subjects’ social value orientation [33], as well as empathy with the Interpersonal Reactivity Index [34] and their susceptibility to external (dis)approval with the Social Desirability Scale [35]. In addition, subjects were asked to indicate their personal income and donation behaviour during the past year. There were no a priori differences in demographic and psychometric variables between the treatment groups (Tables 1 and 2). Moreover, subjects were naive to prescription-strength psychoactive medication and had not taken any over-the-counter psychoactive medication in the preceding 4 weeks. Participants were asked to maintain their regular sleep and waking times and to abstain from caffeine and alcohol intake on the day of the test session. The screenings were conducted prior to the test sessions. The study was approved by the Institutional Review Board of the Medical Faculty of the University Hospital Bonn and was conducted in accordance with the Declaration of Helsinki.

**Table 1:** Demographics, personality traits, and attitudes for CA_high_-scorers.

	OT (n = 10)			PLC (n = 15)				
	Mean		SD	Mean		SD	*t*	P
Age, y	24.20		4.57	26.06		5.11	−0.94	0.36
Education, y	15.56		2.60	17.88		2.53	−2.18	0.04
Donations, EUR/y	24.00		62.22	21.88		39.49	0.11	0.92
Autism-Spectrum Quotient	17.50		5.15	14.94		5.45	1.19	0.25
Childhood-Trauma Quotient	41.10		7.46	40.00		8.08	0.35	0.73
Beck Depression Inventory	2.38		2.07	2.33		2.66	0.04	0.97
State-Trait Anxiety Inventory	33.40		5.48	33.69		6.00	−0.12	0.90
Liebowitz Social Anxiety Scale	11.90		8.99	18.81		16.23	−1.23	0.23
Social Interaction Anxiety Scale	11.60		5.08	16.40		11.22	-1.26	0.22
Social Value Orientation	18.80		1.81	17.60		1.64	1.72	0.10
Social Desirability Scale	21.20		3.08	23.87		1.81	-2.34	0.03
SPF Empathy	39.20		5.05	39.27		6.27	-0.03	0.98

*CA* childhood adversity, *OT* oxytocin, *PLC* placebo, *SD* standard deviation.

Descriptive statistics for participants scoring high on CA (n = 25), shown separately for the OT (n = 10) and PLC (n = 15) groups. The table includes means and standard deviations (SD) for age (in years), years of education, annual donation amounts (in EUR), and scores on various psychometric scales such as the Autism Spectrum Quotient, Beck Depression Inventory, and State-Trait Anxiety Inventory. Between-group differences were analysed using independent-samples *t*-tests, with the resulting *t*-values and two-tailed *p*-values reported for each measure.

**Table 2:** Demographics, personality traits, and attitudes for CA_low_-scorers.

	OT (n = 17)			PLC (n = 12)				
	Mean		SD	Mean		SD	*t*	P
Age, y	25.00		3.86	24.09		2.43	0.70	0.49
Education, y	17.50		3.47	16.41		2.12	0.93	0.36
Donations, EUR/y	23.13		38.29	37.45		51.14	−0.83	0.41
Autism-Spectrum Quotient	16.24		5.64	15.45		4.80	0.29	0.77
Childhood-Trauma Quotient	27.00		2.32	28.64		2.29	−1.83	0.08
Beck Depression Inventory	1.73		2.60	1.00		1.33	0.82	0.42
State-Trait Anxiety Inventory	31.47		5.69	30.27		3.26	0.63	0.53
Liebowitz Social Anxiety Scale	12.41		11.93	10.00		6.37	0.61	0.55
Social Interaction Anxiety Scale	14.47		7.84	13.33		4.96	0.44	0.66
Social Value Orientation	17.82		0.81	17.91		0.51	-0.35	0.73
Social Desirability Scale	20.71		3.00	22.42		3.42	-1.43	0.17
SPF Empathy	40.59		7.18	39.00		5.94	0.63	0.54

*CA* childhood adversity, *OT* oxytocin, *PLC* placebo, *SD* standard deviation.

Descriptive statistics for participants with low CA (n = 29), shown separately for the OT (n = 17) and PLC (n = 12) groups. The table includes means and standard deviations (SD) for age (in years), years of education, annual donation amounts (in EUR), and scores on various psychometric scales such as the Autism Spectrum Quotient, Beck Depression Inventory, and State-Trait Anxiety Inventory. Between-group differences were analysed using independent-samples *t*-tests, with the resulting *t*-values and two-tailed *p*-values reported for each measure.

### fMRI Paradigm

**Fig. 1:**
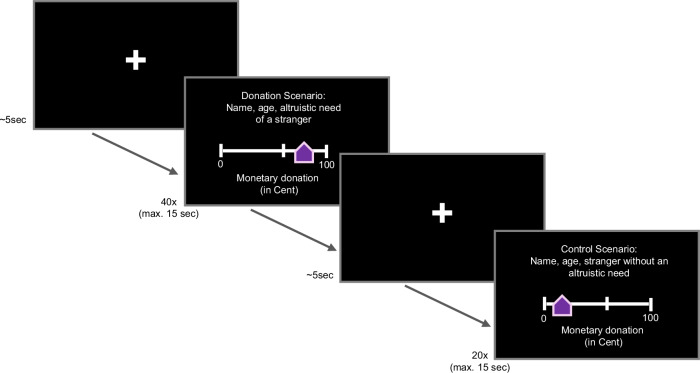
Altruistic donation task design during functional magnetic resonance imaging (fMRI). Participants performed an established one-shot monetary donation task during a 7-Tesla fMRI session, comprising 60 randomized scenarios. Forty scenarios depicted a stranger in need (donation condition), while 20 scenarios showed a stranger without a need (control condition). Each scenario was presented individually, followed by a 5 second interstimulus interval. Participants had up to 15 seconds to indicate their donation using button presses to move a slider displayed on the screen. At the beginning of the task, participants received an endowment of 60 EUR and were instructed that they could donate 0 to 1 EUR per scenario. All donations were deducted from their final payout.

We used an established version of a one-shot, monetary donation task [36] to measure altruistic behaviour (Fig. 1). The task comprised a total of 60 case vignettes: 40 scenarios included a concise description of a person, including their name, age, as well as a personal need and 20 scenarios which described a stranger without a need. These 20 neutral cases served as control items as well as input parameter during the analyses for the neuroimaging data, i.e. donation vs. no donation. All case scenarios were composed in standardized manner and balanced in terms of the age and gender of the needy strangers. Participants had the option to donate 0 to 1 EUR per scenario and could adjust their donation amount in 10 Cent increments on a scale presented below each scenario by pressing two buttons on an MRI-compatible response pad. Prior to the fMRI session, participants were instructed to the donation paradigm using standardized protocols. Furthermore, they were informed that they receive an endowment of 60 EUR, i.e. the maximal amount of donations, and that each donation will be subtracted from this amount. Once subjects were placed in the scanner, they were shown three training trials to practice the task. The scenarios were presented in randomized order, with a 5 second interstimulus interval showing a white fixation cross depicted at the middle of the screen. Participants could proceed to the next scenario either by confirming their donation with a button press or wait for the timeout after 15 seconds. The slider was set to either 0 cents or 100 cents, alternating between the two options, in order to maintain consistency in the effort required to set the donation amount. The donation paradigm was presented on a 32-inch MRI-compatible TFT/LCD monitor (Medres, Cologne, Germany) using Presentation version 14 (Neurobehavioral Systems).

### Image acquisition

A 7-Tesla whole-body MRI research system (Siemens Healthineers) with a 32-channel head array coil (32Rx/1Tx; Nova Medical) was used to obtain T2*-weighted whole-brain images with blood oxygen level-dependent contrast at 1.7-mm isotopic resolution using an accelerated 3D echo planar imaging sequence [37]. Further sequence parameters were: TE/TR = 22 ms/1.9 s, 2 × 2-fold undersampling with GRAPPA reconstruction, 204 mm × 204 mm × 149.6 mm field-of-view with sagittal slice orientation (anterior-posterior primary phase encoding direction). Further scans included gradient echo-based field mapping for geometric distortion correction (3.2 mm isotropic resolution, TA = 54 s) and high-resolution T1-weighted imaging using a GRAPPA 2-fold accelerated MPRAGE with elliptical sampling [38] for anatomical reference (0.6 mm isotropic, TE/TR/TI = 2.91 ms/2.5 s/1.1 s, TA = 6:47 min).

### Statistical analysis

Behavioural data were analysed using SPSS version 24 and 30 (IBM). Quantitative behavioural data were compared using mixed ANOVA and *t*-tests. Based on the subjects’ CTQ scores from the 25-item scale, the sample was median-dichotomized into CA_low_ (n = 29) and CA_high_ (n = 25). To explore whether specific dimensions of CA contributed to the observed effects, we conducted additional exploratory analyses at the level of CTQ subscales (emotional abuse, physical abuse, sexual abuse, emotional neglect, physical neglect). Specifically, we performed a general linear model (GLM) including all five subscales as continuous predictors and their interaction terms with treatment. The neuroimaging data were processed and analysed using the software FSL (Wellcome Centre Integrative Neuroimaging) [39], as well as SPM8 and SPM12 (Wellcome Trust Centre for Neuroimaging) [40] before the data were implemented in MATLAB (MathWorks). Based on the GLM, a two-level random-effects approach was applied. For the first level analyses, two conditions were defined (donation and no donation). Movement parameters and nuisance regressors for physiological noise correction were entered as confounders in the design matrix. In order to facilitate the analysis of the behavioural data in relation to the imaging data, the numerical values of donations were log-transformed, as the data were not normally distributed. Following this transformation, a normal distribution could be demonstrated. The second level analyses were performed by dividing the participants into the following groups: OT + CA_high_, OT + CA_low_, PLC + CA_high_, PLC + CA _low_. We first wanted to assess whether the donation task evoked activation patterns consistent with previous findings e.g., [41–43]. Therefore, we calculated the contrast comparing donations and (neutral) control trials (DON > CON) across all participants of the study. This analysis revealed activity in the prefrontal cortex (two clusters) and the posterior cingulate. The BOLD signal used for the PPI analyses was extracted as the mean time series from spherical regions of interest (6 mm radius) centred on the peak voxels of the clusters identified in the DON > CON contrast and subsequently entered into psychophysiological interaction analyses using the gPPI toolbox [44]. We report only clusters that are significant at the cluster level with family-wise error (FWE) correction and exceed a cluster size of 20 voxels in all analyses. All reported *p*-values are two-tailed and Bonferroni-corrected (Pcorr).

## Results

### Behavioural data

**Fig. 2:**
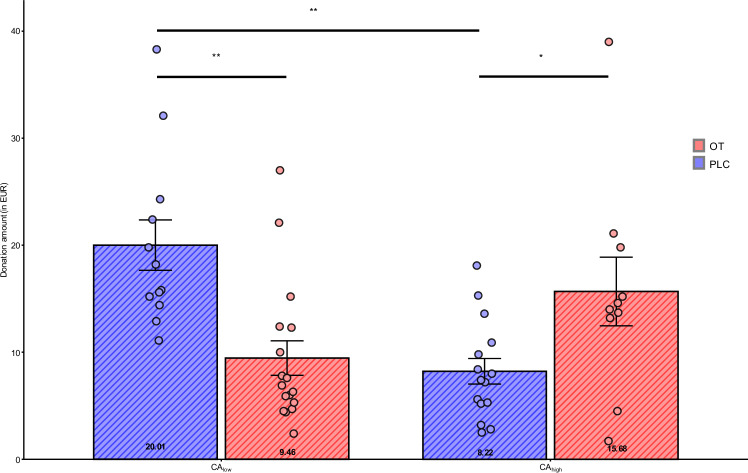
The behavioural effects of OT on altruistic donations are moderated by individual scores in childhood adversity. Among individuals with CA scores above the median, those who received OT (n = 10) donated more money in an altruistic donation task as compared to those who received PLC (n = 15). Among individuals with CA scores below the median, those who received OT (n = 17) donated less money than those in the PLC group (n = 12). Under PLC, the amount donated by the CA low group was more than twice as high as in the amount donated by the CA high group. Error bars represent the standard error. CA childhood adversity, OT oxytocin, PLC placebo. Error bars: SEM; * *p* = 0.02, ** *p* < 0.001.

To investigate the modulatory effect of OT on altruistic donations and CA, a univariate analysis of variance (ANOVA) with CA (low, high) and treatment (OT, PLC) as fixed factors and the donated amount of money as dependent variable was performed. This ANOVA yielded an interaction effect of treatment x CA (F_(1,50)_ = 19.745, *p* < 0.01, *η*^*2*^ = 0.283). Neither the main effect of CA nor that of treatment reached statistical significance (CA: F_(1,50)_ = 1.887, *p* = 0.176, *η*^*2*^ = 0.036; treatment: F_(1,50)_ = 0.581, *p* = 0.449, *η*^*2*^ = 0.011). Among individuals with CA scores below the median (CA_low_), those who received OT donated less money to strangers in need than those in the PLC group (OT: 9.46 EUR ± 6.64; PLC: 20.01 EUR ± 8.15; *p* < 0.001; *d* = 1.447). In contrast, OT promoted an increase in altruistic donations in participants with higher CA scores (CA_high_) (OT: 15.68 EUR ± 10.14; PLC: 8.22 EUR ± 4.63; *p* = 0.02; *d* = −1.022) (Fig. 2). Under PLC, altruistic donations in the CA_low_-group more than doubled those observed in the CA_high_-group (CA_low_: 20.01 EUR ± 8.15 vs. CA_high_: 8.22 EUR ± 4.63; *p* < 0.001; *d* = 1.837). The interacting effects of OT and CA on altruistic donations are further substantiated by a reversal of the correlation between donations and CA as a function of treatment: donations increase with CA under OT but decrease with CA under PLC (CA × Treatment: R^2^ = 0.34, F_(1,51)_ = 26.02, *p* < 0.001). Additional exploratory analyses at the level of CTQ subscales using a general linear model (GLM) revealed a significant interaction between emotional neglect and treatment (F_(1,42)_ = 9.345, *p* = 0.004, *η²p* = 0.182), whereas no other subscale showed a significant interaction.

### fMRI data

**Fig. 3:**
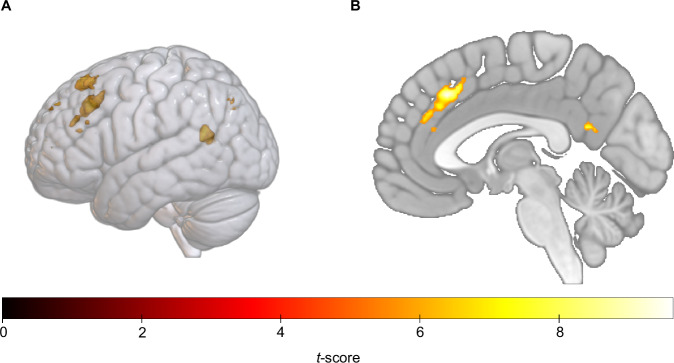
Neural activation associated with the contrast donations > no donations across all participants. **A** FWE-corrected whole-brain results of the second-level analysis. **B** Activation in the mPFC [−2 26 39]. mPFC medial prefrontal cortex, FWE Family-Wise Error corrected.

**Fig. 4:**
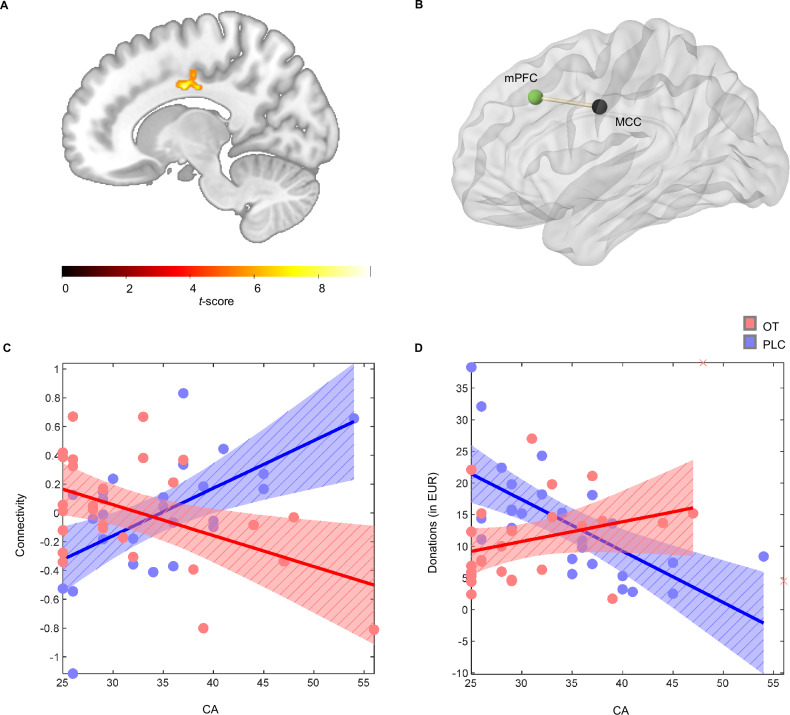
Functional connectivity between mPFC and MCC during altruistic donations. The interacting effects of CA and OT on altruistic donations are reflected in a mirrored effect on functional connectivity between mPFC and MCC: under OT, donations increase, and connectivity decreases with CA; while under PLC, donations decrease, and connectivity increases with CA. **A** Significant Cluster in MCC [13 −7 34]; **B** Representation of the connectivity between mPFC and MCC; **C** Functional connectivity between mPFC and MCC in relation to CA scores (PLC: R^2^ = 0.34, F_(1, 24)_ = 12.25, *p* = 0.0019; OT: R^2^ = 0.23, F_(1, 25)_ = 7.32, *p* = 0.0121; the interaction term used during the analyses was significant, confirming the expected pattern: CA × Treatment: R^2^ = 0.39, F_(1, 52)_ = 33.20, *p* < 0.001); **D** Interacting effects of CA and Treatment (OT; PLC) on altruistic donations (CA × Treatment: R^2^ = 0.34, F_(1, 51)_ = 26.02, *p* < 0.001); CA childhood adversity, MCC middle cingulate, mPFC medial prefrontal cortex, OT oxytocin, PLC placebo.

We observed significant activations in three clusters when comparing donation and control trials (DON > CON) across all participants (Fig. 3A). Two clusters were located in the medial prefrontal cortex (mPFC) at MNI: [−2 26 39] (*t*(53) = 9.74, *p*_FWE < 0.001, *k* = 293) and MNI: [−19 31 58] (*t*(53) = 8.44, *p*_FWE < 0.001, *k* = 146). The third cluster was found in the posterior cingulate (MNI: [−4 −52 20]; *t*(53) = 8.52, *p*_FWE < 0.001, *k* = 100). Given that altruistic decisions are thought to be supported by a network of brain regions e.g., [45], we proceeded to test whether functional connectivity with these three clusters during donation trials changed across treatment groups and as a function of CA. Therefore, generalized psychophysiological interactions (PPI) analyses were performed on the basis of these three clusters as seeds. No significant differences were found between the donations and control conditions in the comparison of the OT and PLC groups, with no clusters surviving the threshold of significance (*p*_FWE < 0.05) and a minimum cluster size of 20 voxels. Further analyses examining high vs. low CA under the same contrast also yielded no significant differences, both in the overall sample and within the PLC group. A flexible factorial ANOVA also showed no significant interaction between the independent variables ‘treatment’ (OT vs. PLC) and ‘CA-group’ (high vs. low) on the contrast DON > CON. However, when we used CA as a continuous independent variable in the PPI analysis, we did find a cluster of voxels whose functional connectivity with one of the mPFC seeds (MNI: [−2 26 39]) changed as a function of CA and treatment, in the middle cingulate (MCC) region (MNI: [13 −7 34]) (Figs. 3B, 4A, B): Connectivity increased with CA under PLC (R^2^ = 0.34, F_(1, 24)_ = 12.25, *p* = 0.0019); while in the OT-group functional connectivity decreased with CA (R^2^ = 0.23, F_(1, 25)_ = 7.32, *p* = 0.0121). This cluster was initially identified in an independent whole-brain SPM analysis using the interaction contrast CA × OT. We then confirmed the observed interaction pattern by extracting and analysing connectivity values from this identified cluster (R^2^ = 0.39, F_(1, 52)_ = 33.20, *p* < 0.001) (Fig. 4C). The interaction between CA and OT on the behavioural level (R^2^ = 0.34, F_(1, 51)_ = 26.02, *p* < 0.001) (Fig. 4D) is reflected in a mirrored effect on functional connectivity between mPFC and MCC (R^2^ = 0.39, F_(1, 52)_ = 33.20, *p* < 0.001) (Fig. 4C): in the OT-group, altruistic donations tend to increase with CA score, while functional connectivity decreases with CA. In contrast, in the PLC group, an increasing CA score is linked to decreasing altruistic donations, while functional connectivity increases. To examine how CA, OT, their interaction and the neural functional connectivity predicted altruistic donation behaviour, we performed a stepwise generalized linear regression on log-transformed donation amounts (Table 3). In Model 1, which included only behavioural predictors, both CA (*b* = –0.08, SE = 0.02, *p* < 0.001) and Treatment (*b* = –3.42, SE = 0.77, *p* < 0.001) were negatively associated with donations. By contrast, the CA × Treatment interaction term showed a positive association (*b* = 0.10, SE = 0.02, *p* < 0.001), indicating that OT mitigated the effect of CA on altruistic behaviour. This finding is consistent with the results of the median-split-based analyses we reported at the beginning of the Results section. This model accounted for 32.2% of the variance in donation amounts (R^2^ = 0.32; adjusted R^2^ = 0.28). Model 2 incorporated both behavioural data and functional connectivity (mPFC – MCC) to examine the combined explanatory power for donation behaviour. While connectivity alone was not a significant predictor (*b* = –0.72, SE = 0.47, *p* = 0.13) its interaction with CA emerged as significant (*b* = 0.04, SE = 0.01, *p* < 0.01). The CA × OT interaction also remained robust (*b* = 0.17, SE = 0.03, *p* < 0.001), indicating additive contributions of behavioural and neural factors. Including functional connectivity alongside behavioural predictors substantially improved model fit, evident in 46.7% of explained variance in donation behaviour (R^2^ = 0.47; adjusted R^2^ = 0.41). A likelihood-ratio test confirmed that Model 2 provided a better fit than the behavioural-only model (*p* < 0.001) (Table 3).

**Table 3:** Generalized linear regression models assessing the extent to which behavioural data (Model 1) and behavioural + functional connectivity data (Model 2) explain altruistic donations.

Model 1					Model 2			
Predictor	b (Estimate)	SE	*t*	P	b (Estimate)	SE	*t*	P
Intercept	5.22	0.64	8.18	<0.001	6.46	0.67	9.64	<0.001
CA, beta^a^	−0.08	0.02	−3.70	<0.001	−0.12	0.02	−5.31	<0.001
OT, beta^a^	−3.42	0.77	−4.43	<0.001	−5.65	0.91	−6.20	<0.001
CA x OT, beta^a^	0.10	0.02	4.08	<0.001	0.17	0.03	6.01	<0.001
Connectivity	n.a.				−0.72	0.47	−1.54	0.13
CA x Connectivity	n.a.				0.04	0.01	2.82	<0.01
R^2^ (ordinary)	0.32				0.47			
R^2^ (adjusted)	0.28				0.41			

*CA* childhood adversity, *OT* oxytocin, *SE* standard error (SE);

^a^regression coefficient.

Both models include predictors CA, OT, and their interaction term (CA × Treatment). Model 2 additionally includes the predictor “Connectivity” and its interaction with CA (CA × Connectivity). Regression coefficients (β) with standard errors (SE), *t*-statistics, and *p*-values are provided for each predictor. Model fit indices (R²) are presented for both the ordinary and adjusted models.

## Discussion

This study investigated how CA and the neuromodulatory effects of OT shape altruistic behaviour. Consistent with prior findings that early adversity may compromise social functioning, increase social threat sensitivity, and reduce prosocial behaviour [3, 5], our behavioural results show that individuals reporting higher levels of CA donated less money to strangers in need under placebo conditions compared to those with lower levels of CA. This supports the notion that early adverse experiences can have long-lasting effects on prosocial behaviour, potentially by altering social dispositions and influencing trust or empathy. Intriguingly, we observed a reversed pattern in participants who received OT: while OT almost doubled altruistic donations in participants with higher CA scores, it reduced donations by 53% in those with lower CA scores (Fig. 2). Notably, although OT increased donations in individuals with higher CA, the donation amounts remained below those observed in low-adversity individuals under PLC, indicating a partial rather than complete restoration of prosocial behaviour. These bidirectional effects align with evidence indicating that OT does not uniformly promote prosocial outcomes [46–48], but that the peptide’s effects are highly sensitive to situational and person-specific factors [9, 13, 49]. This dynamic effect aligns with an inverted U-shaped response, where OT facilitates prosocial behaviour only within an optimal range of social salience sensitivity [14, 50, 51], which is consistent with evidence emphasizing that genetic, neurobiological, and developmental factors contribute to variability in behavioural and neural responses [9, 52]. In CA_high_ individuals, OT may enhance attentiveness to social needs, restoring altruistic behaviour, while in CA_low_ individuals, it may induce oversensitivity, leading to more selective or hesitant responses.

The modulation of functional connectivity between the mPFC and MCC emphasizes the critical role of these circuits in perspective-taking and empathic evaluation - both essential components of altruistic decision-making. The MCC identified here corresponds to the dorsal midcingulate region and is functionally distinct from the dorsal anterior cingulate cortex (dACC), which is more strongly associated with conflict monitoring and cognitive control [53]. Functionally, the MCC and mPFC are key nodes within social-affective networks: the MCC is involved in detecting socially salient or behaviourally relevant stimuli and guiding adaptive responses [54], while the mPFC is centrally implicated in perspective-taking, emotional regulation, and value-based decision-making [55–58]. Our findings should be interpreted in the context of prior work linking childhood maltreatment to alterations in other neural circuits, particularly those involving the amygdala and insula. For example, a recent study demonstrated that emotional abuse is associated with altered amygdala–insula connectivity, a circuit critically involved in affective salience detection and interoceptive processing [59]. In contrast, the mPFC–MCC circuitry identified in the present study is more strongly implicated in higher-order social evaluation, value integration, and action selection. This suggests that CA may impact multiple, partially dissociable neural systems: limbic circuits supporting affective reactivity and threat sensitivity, and medial prefrontal–cingulate circuits supporting social decision-making and prosocial valuation. The results show that under PLC, higher CA scores were associated with increased functional connectivity between the mPFC and MCC - a connectivity pattern that was inversely related to altruistic donations. Notably and consistent with our behavioural data, OT appeared to reverse this pattern by attenuating the connectivity mPFC - MCC in individuals with higher CA (Fig. 4C), while higher CA was also associated with increased altruistic donations (Fig. 4D). By integrating our behavioural and neural data into a predictive regression model (Table 3), we demonstrate that functional connectivity within the social brain - specifically between the mPFC and MCC - modulated by OT, accounts for individual differences in altruistic behaviour, suggesting that altruistic behaviour is not only orchestrated by behavioural traits but is also deeply rooted in the architecture of the social brain. Specifically, our findings indicate that functional connectivity between these regions may not merely support altruistic decision-making, but represent a core mechanism underlying individual differences in prosocial tendencies. Furthermore, OT appears to dynamically recalibrate social salience processing in accordance with early life experiences. This effect is particularly pronounced in individuals with higher CA, in which OT might potentially restore optimal sensitivity to social cues that may have been disrupted by early adversity.

Collectively, our findings contribute to refining theoretical models of the association between CA and prosocial behaviour later in life. While some empirical studies propose that adversity leads to enhanced prosocial tendencies [60, 61], our results align more closely with models that conceptualize CA as variable for diminished prosocial engagement [62]. Given that the self-reported measures of CA we used in our study are somewhat limited, future research adopting more comprehensive assessments spanning chronic, acute, or transient stressors to capture the multifactorial and complex nature of early adversity are warranted. Furthermore, it is important to note that individuals with higher levels of CA, including those in our sample, cannot uniformly be characterized as less altruistic. Rather than supporting categorical distinctions, our findings underscore the need for a more nuanced understanding of the dynamic interplay between early life experiences and neurobiological modulators in shaping prosocial behaviour across varying social contexts. The analyses of CTQ subscales provided initial indications that emotional neglect may be relevant for the observed interaction between CA and OT on altruistic behaviour. Emotional neglect, reflecting reduced emotional support and social attunement during early development stages, has been linked to alterations in socio-affective processing e.g. [63]. However, these findings should be interpreted with caution, as these analyses were exploratory and not specifically powered to detect differential effects across subscales. In addition, the CTQ total score has been shown to provide a more robust and psychometrically stable estimate of overall CA [64, 65], supporting our focus on the total CTQ score in the main analyses. Furthermore, the current study included only healthy young adult males assessed under controlled experimental conditions, which limits the generalizability of the findings to women and to clinical populations with altered social or stress-related functioning. The decision to focus on males was based on evidence showing differences in the prevalence and type of CA between genders, with women more frequently reporting emotional and sexual abuse, and men more commonly exposed to emotional neglect and physical neglect [66]. These distinctions may shape differential neurodevelopmental and behavioural trajectories, including responses to OT. Future research should therefore include more diverse and gender-balanced samples to better characterize the generalizability and boundary conditions of OT’s effects on altruistic behaviour. Although subgroup sample sizes were relatively small, the use of ultra–high-field 7T fMRI provided enhanced spatial resolution and sensitivity, enabling the detection of fine-grained neural effects. Nevertheless, future studies with larger samples will be important to further validate and extend these findings.

In conclusion, this study provides novel insights into the neurobiological mechanisms through which early adversity may shape prosocial behaviour. Specifically, we found that OT influences altruistic donations by implicating changes in functional connectivity in the brain that are central to social-cognitive evaluation and salience processing. These insights not only advance our understanding of how social behaviour is shaped by the interplay between developmental experiences and neuromodulatory systems but also highlight the relevance of individual context in predicting the efficacy of OT-based interventions aimed at enhancing social functioning. As such, this work represents an important step toward a more personalized neurobiological model of prosocial decision-making.

## Data Availability

All data and codes supporting the findings of this study are available upon request. Due to the large size of the 7-Tesla fMRI data, full raw data are not hosted in a public repository but can be made available to researchers under a data-sharing agreement for the purpose of replicating or extending the analyses. All data are available in the main text. Code used for the analyses is available from the corresponding author upon reasonable request.
